# Homeostatic Scaling of Excitability in Recurrent Neural Networks

**DOI:** 10.1371/journal.pcbi.1002494

**Published:** 2012-05-03

**Authors:** Michiel W. H. Remme, Wytse J. Wadman

**Affiliations:** Center for Neurosciences, University of Amsterdam, Amsterdam, The Netherlands; Indiana University, United States of America

## Abstract

Neurons adjust their intrinsic excitability when experiencing a persistent change in synaptic drive. This process can prevent neural activity from moving into either a quiescent state or a saturated state in the face of ongoing plasticity, and is thought to promote stability of the network in which neurons reside. However, most neurons are embedded in recurrent networks, which require a delicate balance between excitation and inhibition to maintain network stability. This balance could be disrupted when neurons independently adjust their intrinsic excitability. Here, we study the functioning of activity-dependent homeostatic scaling of intrinsic excitability (HSE) in a recurrent neural network. Using both simulations of a recurrent network consisting of excitatory and inhibitory neurons that implement HSE, and a mean-field description of adapting excitatory and inhibitory populations, we show that the stability of such adapting networks critically depends on the relationship between the adaptation time scales of both neuron populations. In a stable adapting network, HSE can keep all neurons functioning within their dynamic range, while the network is undergoing several (patho)physiologically relevant types of plasticity, such as persistent changes in external drive, changes in connection strengths, or the loss of inhibitory cells from the network. However, HSE cannot prevent the unstable network dynamics that result when, due to such plasticity, recurrent excitation in the network becomes too strong compared to feedback inhibition. This suggests that keeping a neural network in a stable and functional state requires the coordination of distinct homeostatic mechanisms that operate not only by adjusting neural excitability, but also by controlling network connectivity.

## Author Summary

The central nervous system is continuously adapting to a wide variety of input signals. Single neurons receive from one to thousands of input signals and need mechanisms to prevent their output activity from locking up in quiescence or saturation. One experimentally observed mechanism is homeostatic scaling of neuronal excitability (HSE), which adapts neuronal responsiveness at the time scale of minutes. Most neurons function in networks of excitatory and inhibitory cells. Maintaining stability of activity in such networks is highly relevant, because deviations can result in pathologies like epilepsy. Can HSE control output activity of single neurons without interfering with network stability? To address this question we implement HSE in a neuronal network model. We show that stable functioning of HSE requires that the adaptation rate of the inhibitory cells is slower than that of the excitatory cells. We subsequently investigate various changes in network organization that demand adaptation by HSE, showing that HSE can successfully control activity levels as long as feedback excitation is not stronger than feedback inhibition. This suggests that maintaining stable, functional networks requires the coordination of distinct homeostatic mechanisms, acting not only through adjustments of single cell responsiveness, but also by controlling network connectivity.

## Introduction

Neuronal and synaptic properties exhibit ongoing plasticity during both early development and adult life: neurons show continuous turn-over of ion channels, synapses are formed and eliminated, and existing synaptic connections are altered by processes such as long-term potentiation and depression [1], [2]. At the same time, the firing rate output of a neuron has a limited dynamic working range. Typically neurons are in a quiescent state when input levels are low, whereas the output of the neuron saturates when input levels are high. A neuron can only transmit changes in its input when it functions within its dynamic range, hence, it should avoid both the quiescent and the saturated regime. A neuron can achieve this by employing feedback mechanisms that sense the neuron's activity level and dynamically match its intrinsic excitability to the overall level of synaptic input. Indeed, experiments have demonstrated that neurons regulate membrane properties in response to altered input levels, thereby changing their intrinsic excitability on a time scale of many hours to days [3]–[9]. Recent experiments showed that such homeostatic scaling of intrinsic excitability (HSE) can also occur over tens of minutes [10], [11], suggesting a prominent role in neural functioning on different time scales.

It is often hypothesized that HSE not only serves to keep neurons within their dynamic range, but that it also promotes stability of the local network in which the neuron resides. However, adaptation of intrinsic excitability at the single neuron level could also adversely affect the dynamics at the network level. This is particularly relevant in highly recurrent networks of excitatory and inhibitory neurons, which are ubiquitous throughout the central nervous system. Experimental and theoretical work has illustrated that such networks show a delicate balance between excitation and inhibition for maintaining network stability [12]–[14]. Disturbance of this balance can lead either to quiescence, or to a state in which neurons fire at maximal rates or show synchronized discharges. Hence, also the dynamics at the network level determine whether a neuron can operate within its dynamic range, and importantly, HSE at the single neuron level could interfere with stability of the network.

Here we investigate the requirements for stability of a recurrent network of excitatory and inhibitory neurons showing HSE. We then examine the capacity of HSE to compensate for various plasticity processes in the neural network, and keep the entire network in a stable, functional state, such that all cells operate within their dynamic range. Inspired by the experimental results of [10], [11] we implement HSE as activity-dependent shifts of the neural response function. The shifting of the response functions occurs in such a way that the time-averaged output of a cell remains within its dynamic range. We simulate recurrent networks of excitatory and inhibitory leaky integrate-and-fire neurons that implement HSE. The adapting recurrent network is analyzed using a mean-field approach, describing the activity levels of interacting excitatory and inhibitory neurons at the population level. The mean-field analysis shows the requirements for stable functioning of a HSE controlled recurrent network, thereby also indicating the types of plasticity for which HSE can compensate. Finally, we test the predictions of the mean-field analysis with the spiking network model and illustrate the functioning of HSE for different (patho)physiologically relevant forms of plasticity.

## Results

### Stability requirements for a recurrent network with HSE

**Figure 1:**
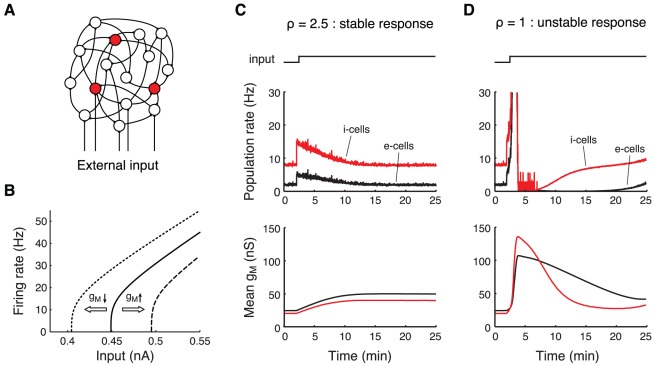
Stable and unstable dynamics of a recurrent network showing HSE. A: Schematic of the network model with e-cells (white circles) and i-cells (red circles) receiving input via recurrent network connections and via excitatory projections from outside the network. B: HSE mechanism operates by shifting the neural response function. The firing rate of an e-cell receiving DC current input is shown (solid line). An increase of the membrane conductance 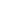 leads to a rightward shift of the response function (dashed line), making the cell less excitable. Decreasing 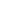 results in a leftward shift of the response function (dotted line), increasing the cell's excitability. C–D: Response of a recurrent network with HSE to a persistent increase in the external drive (top trace) by 50%. In C the i-cells adapt 2.5 times more slowly than the e-cells (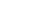). In D both cell types adapt on the same time scale (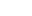). Top panels show the population mean of the instantaneous firing rates of the e-cells (black) and i-cells (red), computed for 1 second bins. Bottom panels show population mean of 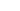 of the e-cells (black) and i-cells (red). The compound external drive to e-cells and i-cells before the input increase is 1.2 kHz.

We constructed a neural network model consisting of populations of recurrently connected excitatory (e-cells) and inhibitory neurons (i-cells), both receiving excitatory input from external sources (figure 1A). The neurons are leaky integrate-and-fire cells to which we added a mechanism that implements HSE. This mechanism uses a slow (calcium-like) variable that signals the recent output activity of the cell. In turn, this slow variable up- or downregulates a membrane conductance 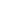 at a time scale controlled by 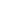 and 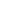 for e-cell and i-cells, respectively. The adaptation time scale is considered to be on the order of tens of minutes. Up- or downregulation of 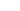 results in, respectively, right- or leftward shifts of the neural response function (figure 1B). The slow variable regulates 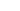 in such a way that the time-averaged output level of the cell remains close to a target value, which we set to 2 Hz for e-cells and to 8 Hz for i-cells (see Methods).

Stability of a network with HSE depends crucially on the relationship between the adaptation time scales of the e-cells and the i-cells. We illustrate the dynamics of a network with HSE in response to a persistent increase in the external drive to both the e-cells and the i-cells (figure 1C–D). The network is initialized in an adapted state where each cell has a time-averaged firing rate that is close to its target value. We define 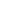 as the ratio of the adaptation time scale of the i-cells to the e-cells (see Methods). First, we consider a network where 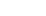, hence the i-cells adapt 2.5 times more slowly than the e-cells (figure 1C). After two minutes of simulated time, the mean external drive to the network is increased by 50%. This causes an instantaneous increase in the firing rates of cells in both populations. To maintain their time-averaged activity levels, both cell types decrease their excitability by slowly increasing 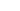 over the course of 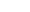 minutes (figure 1C, bottom panel). As a result, the time-averaged activity levels of the cells gradually approach their target levels.

We next set the adaptation time scale ratio 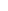 to 1, i.e., both cell types adapt on the same time scale (figure 1D). Following the step increase in the external drive to the network, the network activity again increases instantaneously, and, as a consequence, all cells decrease their excitability (by increasing 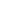) to keep their time-averaged activity levels close to the target levels (figure 1D, bottom panel). However, this now leads to unstable network dynamics with both population rates increasing even further. The activity levels keep increasing, until the network abruptly falls silent, after which the network activity slowly builds up again.

**Figure 2:**
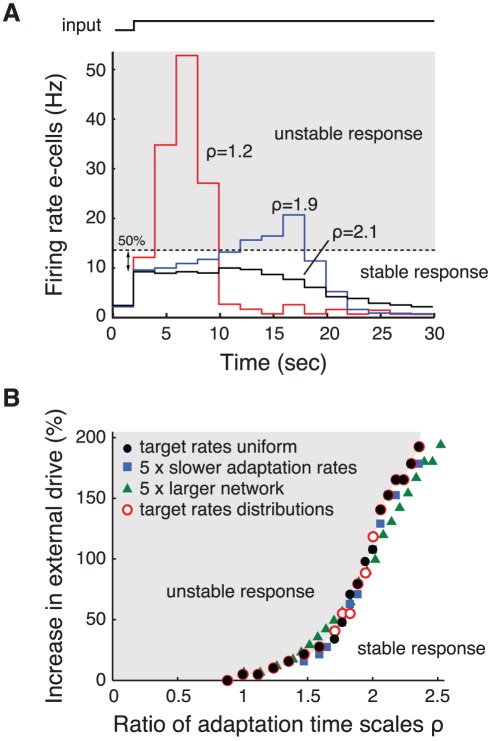
Stable dynamics of HSE controlled networks depends crucially on ratio of inhibitory to excitatory adaptation time scales. A: Response of the excitatory population to a 100% increase of the external drive to both e-cells and i-cells for three different values of 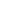, i.e., the ratio of inhibitory to excitatory adaptation time scales. Mean firing rate of the e-cell population is determined in bins of 2 seconds. The response of the adapting network is considered unstable (gray area) when the population rate grows more than 50% compared to the instantaneous increase following the input step. Note that the adaptation rates are 15 times faster than in the simulations shown in figure 1. B: Relationship between 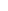 and the maximum increase in external drive that the network can stably adapt to. This relationship is determined for: the standard network (see Methods) where all cells within a population have equal target rates (filled circles); a network where the adaptation rates are slowed down by a factor 5 (squares); a network with 5 times as many neurons (i.e., 4000 e-cells and 1000 i-cells; triangles); and a network in which the target rates of the individual e-cells and i-cells were randomly chosen from a normal distribution with mean 2 and 8 Hz and standard deviation 0.75 and 3 Hz, respectively (open circles). The compound external drive to e-cells and i-cells before the input increase is 1.2 kHz in the 1000 neuron network and 3.1 kHz in the 5000 neuron network.

The ability of the network to adapt in a stable manner to changes in external drive increases with 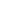. For a range of values of 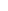, we determined the maximum step in external drive to which the network could adapt without showing the unstable dynamics depicted in figure 1D. In fact, the network shows a gradual transition between stable and unstable responses when 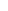 is varied (figure 2A). Hence, we needed to define a criterium for what we consider a stable network response. Upon a step in external drive, the network activity shows an instantaneous increase, which, during a stable response, subsequently decays back to its target level. We define the response as unstable, when, after the initial instantaneous increase, the activity level grows by more than 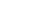 (figure 2A, dotted line). For each value of 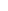, we determined how large the increase in external drive could be without the activity level exceeding this limit (figure 2B, filled circles). The larger 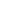 (i.e., the slower i-cells adapt), the larger the increases in external drive that the network can stably adapt to. When i-cells adapt 2 times more slowly than e-cells (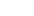), the external drive to the network can increase by 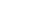
% without the network losing stability. The network is intrinsically unstable when 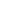 is smaller than a critical value (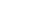); with such small 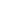, the noise in the network activity is sufficient to produce unstable dynamics as in figure 1D.

We found the same relationship between 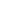 and network stability when varying other key parameters in the network model. Slowing down the adaptation rates for all cells by a factor five, resulted in the same relationship between 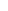 and the maximum input increase (figure 2B, squares), confirming that it is indeed the ratio of the adaptation time scales that is important for the stability of the HSE controlled network. Next we tested whether the relationship is affected by the size of the network model by increasing the number of neurons in the network from 1000 to 5000. Also under these conditions we found the same relationship between 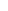 and the maximum input step (figure 2B, triangles). In the above simulations, all cells within a population used the same target activity level. In a final series of simulations we tested the effect of introducing a distribution of target rates within the excitatory and the inhibitory population, using a standard deviation of 0.75 and 3 Hz around the mean target rates of 2 and 8 Hz for e-cells and i-cells, respectively. Again, we observed the same relationship between 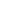 and the maximum increase in external drive (figure 2B, open circles).

**Figure 3:**
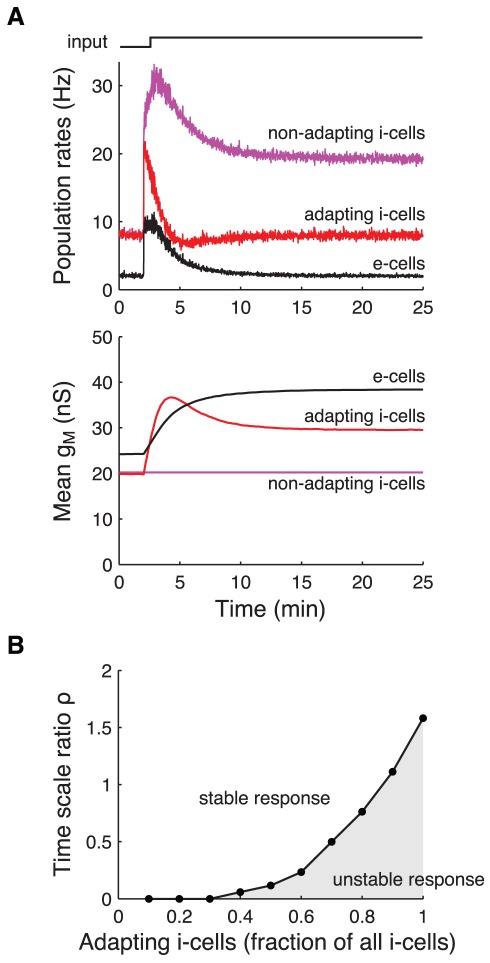
Adapting to a persistent change in external drive to a recurrent network that includes two types of inhibitory cells. A: Response of a recurrent network with two types of i-cells: half of the i-cells show HSE while the other half does not show HSE at the depicted time scale. All cells are perturbed by a 1.2 kHz step increase in the external drive (top trace). Top panel shows the population mean of the instantaneous firing rates of the e-cells (black), the adapting i-cells (red), and the non-adapting i-cells (magenta), computed for 1 second bins. Bottom panel shows the population mean 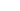 of the three populations. The adaptation time scale ratio 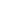 of the (adapting) i-cells to the e-cells is 0.5. The compound external drive to all cells before the input increase is 1.2 kHz. B: Ratio of adaptation time scales 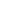 that is required for adaptation dynamics to be stable when the fraction of all i-cells that adapt is varied from 10% to 100%. The network is perturbed from its adapted state (to a 1.2 kHz external drive) by a 0.3 kHz step increase of the external drive to all cells. The same criterium for stability of the adaptation dynamics is used as in figure 2.

Experimental work has demonstrated that i-cells in, e.g., neocortical and hippocampal recurrent networks are very diverse with respect to their intrinsic properties [15], [16]. It is therefore relevant to examine how the stability requirements for a recurrent network with HSE are affected when the network includes multiple populations of different types of i-cells. A detailed examination of such a complex network is beyond the scope of this study. We restrict ourselves to the scenario where the i-cell population consists of two subpopulations that adapt on different time scales. Figure 3A shows the response of the e-cells and the two types of i-cells to a 100% step increase in the external drive to all cells. In this simulation, 50% of the i-cells adapt on a time scale that is two times faster than the e-cells (i.e., 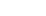), whereas the other 50% of the i-cells do not adapt on the depicted time scale of 25 minutes. In contrast to the results presented above (see figure 2), the network remains stable with such small 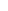 when adapting to such a large perturbation. The time-averaged activity levels of the e-cells (black curves) and adapting i-cells (red curves) reach their target levels through adjustments of their excitability, while the activity level of the non-adapting i-cells (magenta curves) remains elevated.

We next varied the relative size of the two i-cell subpopulations and determined the value of 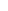 that is required for stable adaptation to a 25% increase of the external drive to all cells (figure 3B). The results show that the smaller the population of adapting i-cells is, the faster they can adapt without destabilizing the adaptation dynamics. No unstable network responses occur when fewer than 30% of i-cells adapt. Hence, a subpopulation of i-cells can adapt much faster than the e-cells, as long as another sufficiently large subpopulation of non-adapting (or, slowly adapting) i-cells guarantees stability of the HSE controlled network.

In summary, stable functioning of a recurrent network with HSE depends crucially on how fast inhibitory cells adapt compared to excitatory cells. The slower inhibitory cells adapt, the more robust the network is to persistent changes in external drive levels.

### Mean-field analysis of a recurrent network with HSE

**Figure 4:**
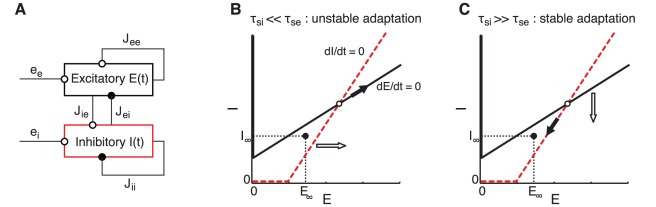
Connectivity and dynamics of the mean-field description of a recurrent network with HSE. A: Scheme of the structure of the network model. The excitatory (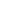) and the inhibitory population (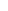) connect to themselves (through 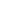 and 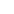) and to each other (through 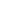 and 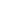). Both populations receive excitatory input from external sources (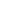 and 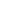). B–C: The 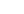-phase plane showing the nullclines 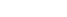 (solid line) and 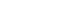 (dashed line). The intersection of the nullclines gives the activity state of the network (open circle). The target state 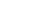 is indicated by the filled circle. Recurrent excitation is strong (i.e., 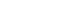), giving the 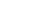-nullcline a positive slope. This strong recurrent excitation can lead to unstable adaptation dynamics, depending on the relative size of the adaptation time scales of the inhibitory population, 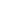, and the excitatory population, 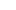 (see equation (3)). Adaptation is unstable (B) when 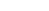: the 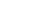-nullcline shifts rightward (open arrow) to decrease excitability of the inhibitory population, thereby moving the network activity state along the 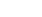-nullcline away from the target state (filled arrow). Adaptation is stable (C) when 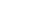: the 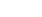-nullcline moves downward (open arrow) to decrease excitability of the excitatory population and the activity state moves along the 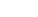-nullcline toward the target state (filled arrow).

To determine the precise requirements for stability of a recurrent network with HSE and the types of plasticity it can cope with, we next analyze the network using a mean-field description. Details of the analysis can be found in the Methods. What follows is a summary of the approach and the main results. We consider a recurrent network consisting of one excitatory and one inhibitory population, both receiving input from external sources (figure 4A). Activity of the excitatory population, 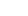, and the inhibitory population, 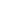, is determined by the excitatory external inputs (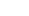 and 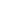, respectively) and by the recurrent interactions in the network through the positive weights 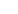, 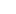, 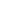, and 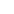. The population activities in the network evolve according to
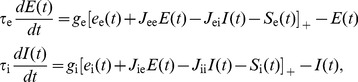
(1)where 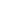 and 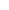 are the time constants of the dynamics of the two populations. To make analysis of the network simpler, we describe the response function of each population by a threshold-linear function 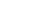, where 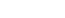 if 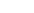, and 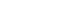 if 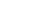, with gains 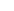 and 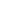 for the excitatory and inhibitory population, respectively. To implement the HSE mechanism, we introduce the variables 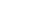 and 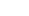. The mechanism operates via activity-dependent shifts of the response functions, analogous to the single cell HSE mechanism in the spiking network model. The HSE mechanism shifts the response functions in such a way that the time-averaged activity levels remain close to the target state (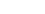), so that both populations function within their dynamic range. The variables 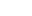 and 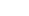 evolve as
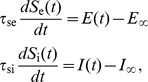
(2)where 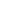 and 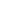 determine the adaptation time scales for the excitatory and the inhibitory population, respectively.

Our aim is to find the conditions for which the target state 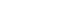 is stable, meaning that perturbations from this state decay to zero. Since the adaptive process described by equation (2) is much slower than the population dynamics that are governed by equation (1), we can consider that equation (1) is always in steady state at the time scale of adaptation. The behavior of equation (1) has been studied many times in the past (see, for example, [12]). Its stability is determined by a pair of conditions that guarantee that inhibition in the network is strong enough to prevent runaway excitation. These conditions do not depend on 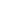, 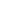, 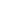 or 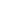. This implies that the activity-dependent shifts of the response function cannot stabilize or destabilize the population dynamics described by equation (1). Assuming that these stability conditions are satisfied, we can now obtain the condition for network stability at the time scale of adaptation:
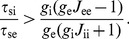
(3)This condition states that the adapting network remains stable when recurrent excitatory coupling is weak (i.e., 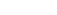), since 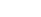 is always positive. However, in a network with strong recurrent excitation (i.e., 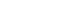), the ratio 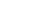 is central to the stability of the adapting network, showing that the inhibitory population needs to adapt sufficiently slow compared to the excitatory population. This corresponds to our findings in the spiking network model, since the ratio 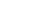 relates directly to the parameter 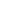. Note that the mean-field model shows an abrupt transition between a stable and an unstable network response when 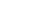 crosses a critical value. This contrasts with the spiking network model, which – due to the nonuniform connectivity – showed a gradual transition when varying 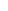 (see figure 2B). Since we consider that the network parameters satisfy the stability conditions for equation (1) (see Methods), we can simplify equation (3) and find that the condition
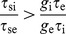
(4)guarantees stability of the adapting network. Hence, when considering that the time constants (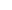 and 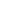) as well as the population gains (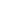 and 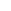) are of similar magnitude, we can state that the adapting network remains stable as long as the inhibitory population adapts more slowly than the excitatory population.

The process by which HSE can destabilize a recurrent network is illustrated in figure 4B. The nullclines 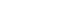 and 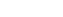 are shown in the 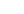-plane. Recurrent excitation is considered to be strong (i.e., 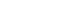) giving the 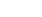-nullcline a positive slope. This strong recurrent excitation can potentially lead to unstable adaptation dynamics, depending on the relative adaptation time scales 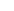 and 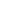. The activity state of the network lies at the intersection of the nullclines (open circle). The network activity has been perturbed from the target state (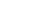) (filled circle) such that 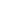 and 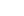 are larger than 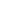 and 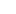, respectively. It is easiest to see how relatively fast adaptation of the inhibitory population destabilizes the network by considering that 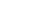. Because 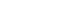, the 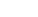-nullcline shifts rightwards (open arrow), corresponding to a decrease of the excitability of the inhibitory population. However, as a consequence the network state 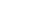 moves along the 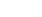-nullcline, away from the target state. The increase of 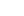 leads to further rightward shifts of the 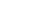-nullcline and consequently to further increases of the network activity. Hence, the network is destabilized when adaptation of the inhibitory population is fast compared to that of the excitatory population. In contrast, figure 4C demonstrates that the network remains stable when the excitatory population adapts faster than the inhibitory population (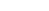). Now the 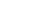-nullcline shifts downwards to decrease the excitability of the excitatory population (open arrow), and as a result, the network state moves toward the target state (filled arrow).

Summarizing, the mean-field analysis of adapting recurrent networks shows that HSE can keep the time-averaged activity levels of the neural populations within their dynamic range. HSE achieves this for any type of network plasticity that does not destabilize the fast population dynamics. Stability of the adaptation dynamics itself depends critically on the relationship between the adaptation time scales of the two neuron populations combined with the gains of their respective response functions.

### HSE keeps cells within their dynamic range for various forms of plasticity

The previous sections were focussed on the requirements for HSE to function stably in a recurrent network. Considering that the purpose of HSE is to keep the time-averaged activity of cells within their dynamic range in the face of ongoing plasticity, we next examine the response of the spiking network model to various (patho)physiologically relevant types of plasticity. We first determine the response of both a non-adapting network and a network with HSE to varying levels of external drive. Subsequently we focus on three changes in network parameters that affect the excitation-inhibition balance. The balance between the recurrent excitatory and inhibitory input is crucial to network stability as many theoretical studies have demonstrated in the past [14], [17]–[20]. We determine how the various parameters affect the network activity, to what degree a network with HSE can adapt to changes in these parameters, and how these parameters affect the requirements for stable adaptation dynamics.

#### Adapting to persistent changes in external drive

First, we consider the steady state response of a recurrent network to a persistent change in the external drive. Note that the level of external input is expressed in the compound external drive to each cell (in kHz). Different levels of this external drive can represent both changes in the frequency of the external input as well as changes in the number of external inputs that project to the cells.

**Figure 5:**
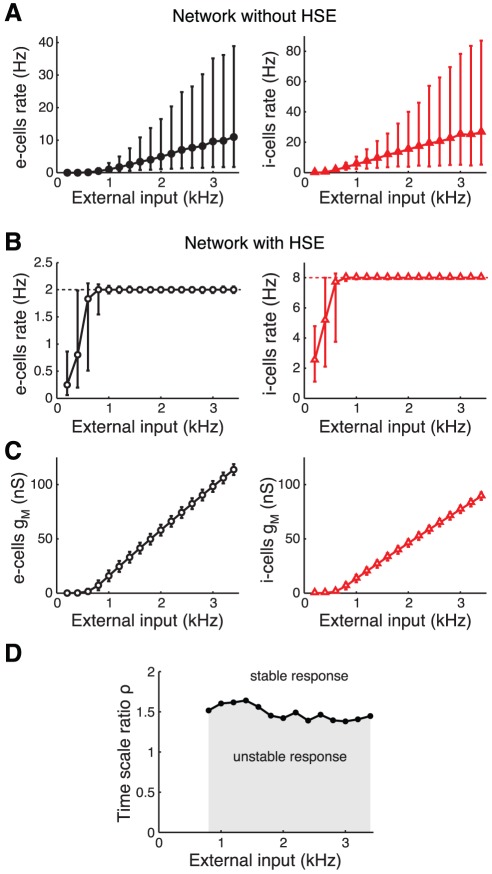
Adapting to persistent changes in external drive to the recurrent network. Response of a non-adapting network and a network with HSE to a range of external excitatory input levels to both e-cells and i-cells. Since the number of external inputs and their frequency are interchangeable in our model, the external excitatory drive is expressed in the compound rate (in kHz). A: Median firing rate of e-cells (left) and i-cells (right) in a network without HSE. Error bars give 5%–95% percentiles. B: As in panel A for network with HSE. Dotted lines at 2 Hz (left) and 8 Hz (right) give target rates of e-cells and i-cells. C: Population mean 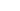 of the e-cells (left) and i-cells (right) in the network with HSE. Error bars give standard deviation. D: Required ratio of adaptation time scales 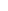 for stable adaptation dynamics when the network is perturbed from its adapted state characterized in panels B and C. The perturbation consists of a 0.3 kHz step increase of the external drive to all cells. The same criterium for stability of the adaptation dynamics is used as in figure 2.

The non-adapting network shows an approximately linear relationship between the output of both the e-cells and i-cells and the external drive (figure 5A). The firing rates show a wide distribution (median and 5%–95% percentiles), because the number of recurrent inputs that the cells receive is variable (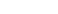 mean and standard deviation). The large variability in firing rates reflects that the neural output is very sensitive to the balance of the excitatory and inhibitory input that a neuron receives: some neurons are quiescent and others are highly active, while the number of inputs to each neuron differs by only a few percent. This emphasizes the need for homeostatic control of neural activity in a recurrent network.

In the network with HSE, the time-averaged activity levels of all neurons are indeed controlled over a wide range of external input levels (figure 5B). The control of the intrinsic excitability in the HSE network occurs via up- and downregulation of the membrane conductance 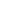. The population mean 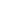 of the e-cells and the i-cells of the adapted network shows a linearly increasing relation with the external drive (figure 5C). The variability in the number of recurrent inputs that each neuron receives leads to a narrow distribution of 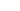. For low compound external drive (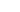 kHz), the population mean 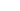 of the e-cells and the i-cells becomes very small, making the cells maximally excitable such that they can produce their target firing rate output. However, when the external input level becomes too low and 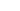 approaches zero, more and more cells cannot reach their target activity levels anymore. In contrast, for high external input levels the cells can, in theory, always decrease their excitability sufficiently by increasing 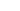. When assuming that 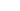 cannot increase further than 150 nS, the maximal compound external drive for which the network can adapt is 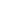 kHz.

The different levels of external drive to the network do not affect the stability of the adaptation dynamics when the network receives further perturbations (figure 5D). We determined the ratio of adaptation time scales 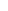 that is required for stable adaptation dynamics when the network is perturbed from its adapted state (characterized in figure 5B–C). For all levels of external drive to the network, a 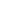 of at least 1.4 to 1.6 is required for stable adaptation to the perturbation, which consists of a 0.3 kHz step increase of the external drive to all cells in the network.

In summary, for a wide range of external input levels, HSE can keep the entire recurrent network in a functional state where all cells operate within their limited dynamic range.

#### Adapting to persistent changes in the external drive to excitatory cells

In the above section, the ratio of excitatory to inhibitory input that each cell receives, changed little when varying the level of external input, since the external input projects to both the e-cells and the i-cells. However, external inputs to a local neural network often target specific cell types (see, for example, [21]). Plasticity in the number of these inputs (or, equivalently in our model, persistent changes in the activity of these presynaptic neurons) can affect the excitation-inhibition balance in the local network. We examine this with the spiking network model by varying the compound external drive to the e-cells, while keeping the compound external drive to the i-cells constant.

**Figure 6:**
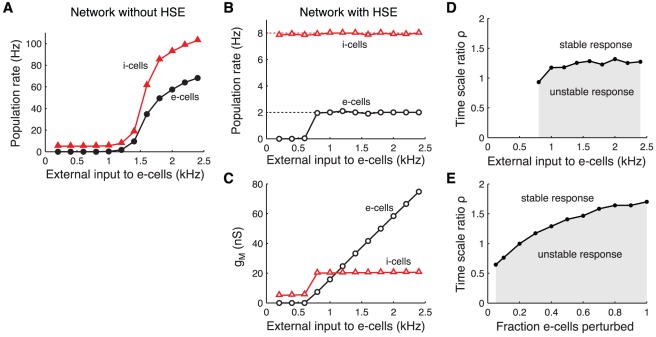
Adapting to persistent changes in the external drive to excitatory cells. Steady state response of the recurrent network when the compound external excitatory drive to the e-cells ranges from 0.2 to 2.4 kHz, while the external drive to the i-cells is constant at 1.2 kHz. A: Median firing rate of e-cells (circles) and i-cells (triangles) in the network without HSE. B: Median firing rate of e-cells (circles) and i-cells (triangles) in the network with HSE. Dotted lines at 2 and 8 Hz give target rates of both cell types. C: Population mean 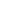 of e-cells (circles) and i-cells (triangles) in the network with HSE. D: Required ratio of adaptation time scales 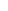 for stable adaptation dynamics when the network is perturbed from its adapted state (characterized in panels B and C). The perturbation consists of a 0.1 kHz step increase of the external drive to the e-cells. The same criterium for stability of the adaptation dynamics is used as in figure 2. E: As in D, except that a perturbation of 0.3 kHz is applied to 5% to 100% of the e-cells. The perturbations are applied to a network that is adapted to a compound external input level of 1.2 kHz.

Varying the drive to e-cells has strong effects on the activity of the non-adapting recurrent network (figure 6A). A 50% increase in the external drive to the e-cells from 1 kHz to 1.5 kHz leads to a large increase in e-cell activity from 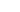 Hz to 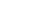 Hz. In turn, the e-cells also drive the i-cells to high activity levels. In contrast to the non-adapting network, the time-averaged activity levels of both e-cells and i-cells in the network with HSE remain close to their target rates over a wide range of compound external input levels (figure 6B). As the drive to the e-cells increases, the excitability of the e-cells decreases (i.e., 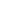 grows, figure 6C). The homeostatic mechanism only fails when the external drive to the e-cells is so low (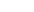 kHz) that 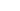 approaches zero and the e-cells cannot increase their excitability any further to reach the target activity level.

Different levels of external drive to the e-cells have little effect on the stability of the adaptation dynamics (figure 6D). To adapt to a 0.1 kHz step increase of the external drive to the e-cells, a 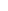 of at least 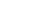 is required for most levels of external drive to the e-cells. The time scale ratio 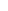 can be smaller when only a fraction of the e-cells is perturbed (figure 6E). However, even when only 5% of the e-cells receive a perturbation consisting of a 0.3 kHz step increase in external drive, the i-cells can adapt at most 50% faster than the e-cells.

Thus, changes in the excitation-inhibition balance resulting from changes in the external drive can have strong effects on network activity levels. However, HSE can largely control for such plasticity and maintain a functional network state.

#### Adapting to plasticity of the recurrent excitatory connection strength

Plasticity of the strength of recurrent excitatory connections is another biologically relevant source of changes in the excitation-inhibition balance in a network. This can result from learning processes, such as spike timing-dependent plasticity [22], [23]. We varied the strength of all recurrent excitatory connections (equivalent to 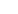 in the mean-field model) in the spiking network model and determined the resulting state in a network with or without HSE. Note that under more realistic conditions, synaptic plasticity is expected to result in increases of some synaptic weights, while other weights decrease. As a consequence, part of the effects on the excitation-inhibition balance would cancel.

**Figure 7:**
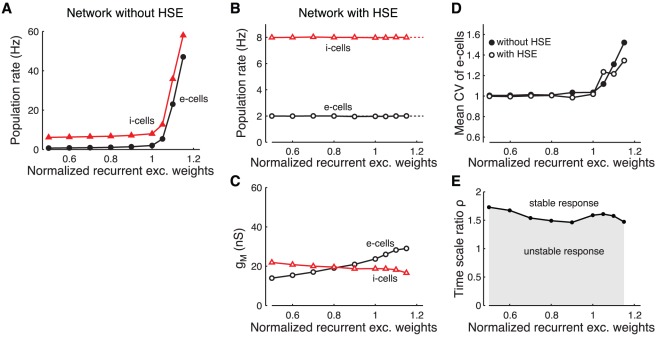
Adapting to plasticity of the recurrent excitatory connection strength. Response of the recurrent network when varying the normalized strength of the recurrent excitatory connections from 0.5 up to 1.15 (see Methods for the standard values). A: Median firing rate of e-cells (circles) and i-cells (triangles) in the network without HSE. B: Median firing rate of e-cells (circles) and i-cells (triangles) in the network with HSE. Dotted lines at 2 and 8 Hz give target rates of both cell types. C: Population mean 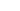 of the e-cells (circles) and i-cells (triangles) in the network with HSE. D: Population mean coefficient of variation of the interspike intervals (CV) of the e-cells in the network without HSE (filled circles) and in the network with HSE (open circles). E: Required ratio of adaptation time scales 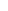 for stable adaptation dynamics when the network is perturbed from its adapted state (characterized in panels B and C). The perturbation consists of a 0.3 kHz step increase of the external drive to all cells in the network. The same criterium for stability of the adaptation dynamics is used as in figure 2.

Activity levels in the non-adapting network rise steeply when increasing the recurrent excitatory synaptic weights above their standard values (figure 7A; see Methods for standard values). Decreasing the recurrent excitatory weights causes a gradual decrease of the network activity levels. The HSE network is able to maintain the time-averaged output levels for decreasing values of the recurrent excitatory synaptic weights (figure 7B), which requires only moderate changes in cell excitability (figure 7C). The HSE mechanism also controls the time-averaged output levels for limited increases of the recurrent weights. However, either with or without HSE, the network becomes gradually more unstable when the normalized excitatory weights are increased from 1 to over 1.15, and starts showing highly synchronized discharges. We quantified the irregularity of spiking activity in the network by calculating the coefficient of variation (CV) of the interspike interval (ISI) distribution of the e-cells (figure 7D). When recurrent excitatory weights are set to their standard value (or smaller), the mean CV is close to 1 in both the HSE controlled network and in the network without HSE, reflecting the irregular, Poisson-like firing statistics. When the recurrent excitatory weights are increased, synchronized discharges occur increasingly often, which is expressed in the rising CV. While HSE can control the time-averaged activity levels, it cannot prevent these synchronized discharges from occurring, hence the activity fluctuates between low and high activity levels. These results agree with the mean-field analysis, which showed that HSE cannot compensate for parameter changes that make recurrent excitation too strong compared to feedback inhibition; this destabilizes the population dynamics determined by equation (1).

Stability of the adaptation dynamics is largely independent of the strength of the recurrent excitatory weights (figure 7E). The adaptation time scale ratio 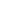 needs to be larger than 1.5 when the network adapts to a 0.3 kHz step increase of the external drive to all cells in the network. Note that even when the weights are decreased by 50%, recurrent excitation is still very strong and as a consequence the adaptation dynamics still become unstable when i-cells adapt too fast.

Summarizing, HSE is able to control activity levels in a recurrent network for decreasing strength of the recurrent excitatory connections. However, it cannot prevent the loss of stability that results when the strength of the recurrent excitatory connections becomes too large.

#### Adapting to loss of inhibitory cells

A third important parameter determining the excitation-inhibition balance in a network is the ratio of the number of e-cells to i-cells. This ratio can change over time because of pathological conditions, for example, the decrease in the number of inhibitory cells resulting from the cell-type specific cell loss in certain forms of epilepsy [24], [25]. In this final section, we examine the network response to a decrease in recurrent inhibition resulting from the removal of i-cells from the network.

**Figure 8:**
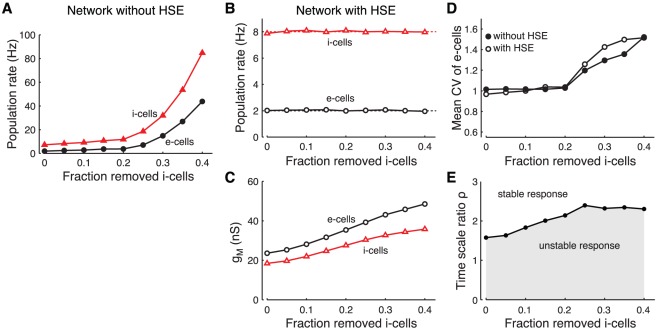
Adapting to loss of inhibitory cells from the recurrent network. Response of the recurrent network when up to 40% of the inhibitory cells are removed. A: Median firing rate of e-cells (circles) and i-cells (triangles) in the network without HSE. B: Median firing rate of e-cells (circles) and i-cells (triangles) in the network with HSE. Dotted lines at 2 and 8 Hz give target rates of both cell types. C: Population mean 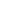 of the e-cells (circles) and i-cells (triangles) in the network with HSE. D: Population mean coefficient of variation of the interspike intervals (CV) of the e-cells in the network without HSE (filled circles) and in the network with HSE (open circles). E: Required ratio of adaptation time scales 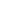 for stable adaptation dynamics when the network is perturbed from its adapted state (characterized in panels B and C). The perturbation consists of a 0.3 kHz step increase of the external drive to all cells in the network. The same criterium for stability of the adaptation dynamics is used as in figure 2.

Removing up to 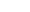 of the i-cells in the non-adapting network has little effect on activity levels (figure 8A). However, the population activity levels increase rapidly when larger fractions of i-cells are removed. In the network with HSE, the time-averaged firing rates remain under control as an increasing fraction of i-cells is removed from the network (figure 8B). The e-cells and the remaining i-cells accomplish this by gradually decreasing their excitability (figure 8C). However, when more than 20% of the i-cells is removed, both the HSE controlled network and the network without HSE become gradually more unstable on the fast time scale of the population dynamics and start showing synchronized discharges. These transitions between low and high activity levels are reflected in the increased CV of the e-cell ISI distributions (figure 8D). Hence, while HSE can still control mean activity levels when more than 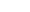 of i-cells are removed, it cannot avoid the occurrence of the synchronized discharges. These results agree with the mean-field analysis in which a decrease in the number of i-cells corresponds to a decrease in the weights 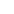 and 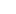. When feedback inhibition becomes too small compared to recurrent excitation, the network becomes unstable at the time scale of the population dynamics described by equation (1), and this instability cannot be counteracted by HSE.

Finally, the fewer i-cells are remaining in the network, the slower they need to adapt to keep the adaptation dynamics stable (figure 8E). When perturbing all cells in the network with a 0.3 kHz step increase of the external drive, the adaptation time scale ratio 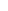 needs to be at least 1.5 when no i-cells are removed from the network, and increases up to 2.3 when 40% of the i-cells are removed.

## Discussion

Recent experimental studies demonstrated activity-dependent scaling of intrinsic excitability in cortical and hippocampal excitatory and inhibitory neurons [3]–[11]. It is generally thought that this process serves to compensate for the ongoing functional and morphological plasticity in neuronal and synaptic properties such that the intrinsic excitability remains matched to the synaptic input level. In this way, homeostatic scaling of intrinsic excitability (HSE) ensures that neurons operate within their limited dynamic range, slowly adapting to specific average input levels, while keeping up a high sensitivity to changes in synaptic input. In addition, this process is thought to promote network stability. Here, we explored the consequences of HSE implemented in single cells on the stability of recurrent networks, and the ability of HSE to facilitate adequate responses in a network that undergoes several forms of long-lasting plasticity.

Experimental and theoretical work has demonstrated that stability of recurrent networks requires a delicate balance between excitation and inhibition [12]–[14]. A central issue in our study was to determine the requirements for stable functioning of HSE in such recurrent networks. Simulations with a model consisting of spiking excitatory and inhibitory cells that implement HSE demonstrated that the ratio of adaptation rates of the two cell types is the key parameter determining the stability of HSE controlled networks (figure 1C–D). The slower inhibitory cells adapt, the larger the persistent changes in external drive to which the network can adapt (figure 2). A mean-field model describing the activity levels of interacting excitatory and inhibitory populations allowed a mathematical analysis of the stability of a network with HSE. The results confirmed that stability of the adapting network critically depends on the ratio of the adaptation rates of both populations. Assuming that the population gains and the time constants of the population dynamics are similar, stability of the adapting network is guaranteed when the inhibitory population adapts slower than the excitatory population.

With the mean field model we could also demonstrate the process by which HSE can destabilize a recurrent network (figure 4B). In our model a HSE-induced decrease in excitability of the inhibitory population paradoxically leads to increases in its activity level. This process is closely related to a phenomenon described by Tsodyks and colleagues [26], who showed that inhibiting the inhibitory population in a recurrent network can result in an increase of the inhibitory population activity. In our HSE controlled network model, the ongoing adaptation reinforces these changes in population activity levels, consequently leading to unstable dynamics on the time scale of the homeostatic process.

The above requirements for stable adaptation dynamics are relaxed somewhat if the inhibitory population consists of multiple subpopulations of cells that adapt on different time scales. The adapting network can then remain stable when a subpopulation of inhibitory cells adapts faster than the excitatory cells, as long as there is another sufficiently large subpopulation of inhibitory cells that adapts more slowly (figure 3). To our knowledge, no experimental studies have assessed the relative adaptation time scales of excitatory cells and the various types of inhibitory cells that have been described in recurrent networks [15], [16]. In fact, only few studies have examined the homeostatic changes in intrinsic excitability of inhibitory cells when network activity levels are manipulated [4], [6], [9]. Those studies demonstrated that two inhibitory subtypes in cortical networks (parvalbumin-positive and somatostatin-positive neurons) indeed show HSE. Our results suggest that it is important for future experimental work to examine the relative adaptation time scales of the various cell types in recurrent networks. This could be particularly relevant for pathological conditions in which networks show abnormal dynamics, e.g., various forms of epilepsy (see also below).

Once we defined the requirements for stable functioning of HSE in recurrent networks, a key question in this study was to pinpoint forms of plasticity where HSE could be of relevance and guarantee that neurons function within their dynamic range. The results from the mean-field analysis imply that HSE can realize control for any change in network parameters as long as these changes do not destabilize the network dynamics described by equation (1): the conditions that determine the stability of the network at this fast time scale are not controlled by HSE (equations (9) and (10)). Simulations with the spiking network model showed that HSE can indeed adapt to persistent changes in the external drive to the network (figure 5). The ability of HSE to compensate for plasticity is particularly important when considering changes that affect the excitation-inhibition balance in the network. The results showed that such changes have strong effects on the activity of a non-adapting recurrent network. We tested three biologically relevant sources of such plasticity. First we showed that HSE can adapt the network to persistent changes in external drive to only the excitatory cell population (figure 6). Such gross modifications in input strength will be encountered as an increase in total synaptic input during development, but also as a decrease in the number of synaptic inputs during degenerative diseases. We then showed that HSE can also cope with variations in synaptic strength that could occur under normal physiological conditions (figure 7). As a final example we illustrated how HSE can control cell activity when (sub)classes of cells are eliminated, a situation that is encountered in specific forms of epilepsy (figure 8). These examples also illustrated that HSE will never prevent network instabilities that occur when recurrent excitatory connectivity becomes too strong, or for the same token, when the strength of inhibitory feedback in the network becomes too weak.

The analytical as well as numerical results suggest that, in order to compensate for ongoing plasticity, recurrent networks need, in addition to HSE, mechanisms that control connectivity (e.g., the number of inputs per cell) and synaptic efficacy (e.g., the mean synaptic efficacy of inputs to the cell). Indeed, experiments have demonstrated that the ratio of the number of excitatory and inhibitory synapses onto pyramidal neurons is maintained [27], [28]. Also, a series of studies have demonstrated a mechanism that regulates the overall efficacy of the synapses impinging on a single cell, a process known as synaptic scaling [29], [30]. Like HSE, synaptic scaling has been hypothesized to serve as a homeostatic control mechanism for maintaining neural activity levels to keep cells within their dynamic range. There are indications that synaptic scaling is particularly relevant during development and acts on a very slow time scale (hours to days). A key difference with HSE is that in order to preserve the relative synaptic weights of all synapses, synaptic scaling needs to be proportional for all thousands of synapses over the dendritic tree of a neuron. This is important as it is assumed that information is stored in the relative weights of the synapses. The HSE mechanism in our study that acts via up- and downregulation of ion channels that control excitability, avoids this problem. It has not yet been investigated in detail how synaptic scaling interferes with network stability (but see [31]). It is important for future studies to determine how the various homeostatic control mechanisms operate together and maintain a functional neural network (for review, see [32]).

As a concluding remark, we want to emphasize that our results suggest that a defect in the HSE mechanism can have severe consequences for network functioning. For example, when excitatory neurons cannot adapt effectively (e.g., because of a defect in the molecular mechanism underlying HSE) the network can become unstable or be in a state that is close to instability. Possibly such defects are involved in certain pathologies, such as epilepsy. It is important to realize that such defects are not necessarily observable in either abnormal cell excitability or network connectivity, but rather in the adaptation dynamics that accompany ongoing plasticity. This suggests that it is important to consider the adaptation properties of cells as a possible source of the abnormal network dynamics seen in several pathologies.

## Methods

### Network model of spiking neurons

#### Network

The network parameters are based on a model described in [33] that consists of 4000 e-cells and 1000 i-cells with 20% connection probability between any pair of cells. In most simulations, we scale the number of neurons down by a factor five to reduce simulation time, leading to a network of 800 e-cells and 200 i-cells. The connection probability and weights of the connections are adjusted according to rules derived by [34] to compensate for the decrease in the number of connections in the smaller network. This gives a connection probability of 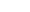 for all four connection types: e-cells to e-cells, e-cells to i-cells, i-cells to e-cells and i-cells to i-cells. In addition to the 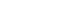 recurrent inputs, the neurons also receive external excitatory input consisting of independent Poisson spike trains with a given mean rate. The default compound external drive to each neuron is 1.2 kHz.

#### Neurons

The neurons are modeled as leaky integrate-and-fire neurons (LIF). The membrane potential 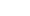 of neuron 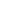 evolves according to
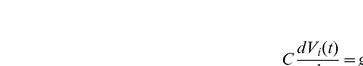
with membrane capacitance 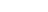 nF for the e-cells and 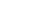 nF for the i-cells. The total excitatory synaptic conductance 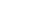 with reversal potential 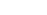 mV consists of the sum of all excitatory synaptic conductances to neuron 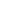 (see below). Equivalently, the total inhibitory synaptic conductance 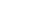 with reversal potential 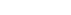 mV consists of the sum of all inhibitory synaptic conductances to neuron 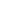. The spike threshold is 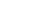 mV, the reset potential is 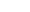 mV, and the absolute refractory period is 2 ms for e-cells and 1 ms for i-cells. The voltage-independent membrane conductance 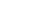 with reversal potential 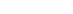 mV is a variable in many of our simulations (see below) and represents the sum of the various subthreshold conductances that neurons possess. When simulating non-adapting networks, 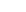 is set to 25 nS for e-cells and 20 nS for i-cells, yielding membrane time constants of 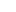 ms and 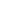 ms, respectively.

#### Synapses

The excitatory AMPA-type synapses and the inhibitory GABAergic synapses are modeled as conductance changes that activate with a certain delay after the presynaptic spike. The delays are determined by a uniform distribution between 0 and 2 ms. The time courses of the conductance changes are modeled as the difference of two exponential functions (see, for example, [33]). The rise and decay times are 0.5 and 2 ms, respectively, for the AMPA synapse, and 0.5 and 5 ms, respectively, for the GABA synapse. The peak conductances of both types of synapses are taken from [33] and are scaled for simulations with the smaller network of 1000 neurons (see above) according to the rules derived in [34]. The values for the AMPA synapse are 3.8 nS (external input to e-cells), 3.1 nS (external input to i-cells), 2.9 nS (e-cells to e-cells) and 2.3 nS (e-cells to i-cells). For the GABA synapse the values are 18.9 nS (i-cells to e-cells) and 15.1 nS (i-cells to i-cells). At a membrane potential of 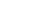 mV these peak conductances lead to excitatory and inhibitory postsynaptic potentials of 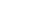 mV and 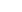 mV, respectively.

#### HSE mechanism

A mechanism is added to the LIF neurons to implement the homeostatic scaling of intrinsic excitability. The mechanism attempts to keep a neuron within its dynamic range by keeping the time-averaged output activity close to a value that is within the neuron's dynamic range. Experiments in hippocampal and cortical networks demonstrated that HSE typically leads to changes of the input resistance and shifts of the neural response function, while having little effect on the resting membrane potential [6], [7], [9]–[11]. Inspired by these results, we implement HSE as an activity-dependent regulation of the total subthreshold membrane conductance 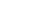. Varying 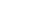 results in shifts of the neural response function (see figure 1B, and, for example, [35]), and determines the input resistance. We use a mechanism for regulating 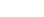 that is closely related to mechanisms proposed in [36], [37] for regulating ion conductance densities in order to maintain specific firing patterns. A slow variable 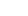, which could reflect an intracellular calcium concentration [38], is added to the LIF neurons and evolves as
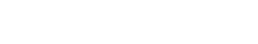
where 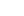 denotes the Dirac delta function. The equation implies that with every spike produced by neuron 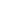, occurring at time 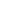, the slow variable 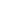 is increased by a value 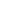. In between spikes 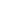 decays exponentially with time constant 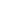. Inspired by experimental data on calcium dynamics, we set the values 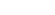 ms and 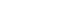 nM [39]. The dynamics of 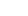 lead to a linear relation between the time-averaged firing rate of neuron 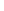 and the value of 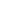. Hence, we can now define a target value 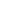 that is related to the target time-averaged firing rate of neuron 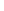. The conductance density 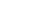 is upregulated when 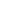 is larger than 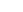 and downregulated when 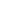 is smaller than 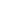:
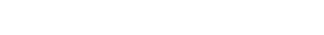
where the function 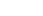 serves to bound 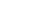 between 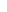 nS and 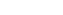 nS. For 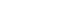 we have 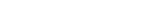, and otherwise 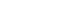. The time scale of adaptation for the cells is determined by the constants 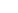 (for the e-cells) and 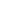 (for the i-cells) and the function 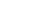. We define the ratio of the adaptation time scales of i-cells to e-cells as 
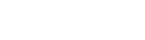
, where 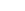 is the population average 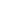 of the e-cells, and 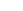 is the population average 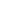 of the i-cells.

On the basis of data from [10], [11], the adaptation time scales should be such that adaptation evolves at the time scale of tens of minutes to hours. It is important to realize that the network activity will reflect input variations that occur on shorter time scales. In many simulations we decrease 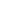 and 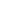 by a factor 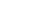 to reduce simulation time, while making sure that this does not affect the results (see figure 2B). In most simulations we set the target time-averaged firing rates of the excitatory and the inhibitory neurons to 2 and 8 Hz, respectively, based on the typical low mean firing rates recorded in cortical neurons *in vivo* (see, for example, [40], [41]).

### Mean field analysis of network showing HSE

We analyze the dynamics of an adapting recurrent network that consists of an excitatory and an inhibitory population that both receive excitatory input from external sources. Using a mean-field approach we describe the average activity of the cells within each population. The activity of the excitatory population 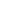 and the inhibitory population 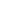 is determined by the excitatory external inputs 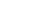 and 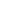, respectively, and by the recurrent interactions in the network through the positive weights 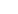, 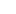, 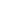, and 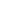 (see figure 4A). Assuming first order kinetics, the population activities in the network evolve according to
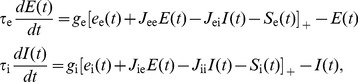
(5)where 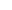 and 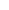 are the time constants of the dynamics of the excitatory and the inhibitory population, respectively. The response function of each population is described by a threshold-linear function 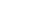, where 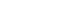 if 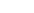, and 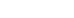 if 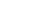, with gain 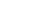 for the excitatory population and 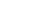 for the inhibitory population. The variables 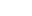 and 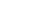 are introduced to implement the HSE mechanism (performing the same role as 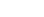 in the spiking network model), operating via activity-dependent shifts of the response functions. We assume that the HSE mechanism shifts the response functions in such a way that the time-averaged network activity levels remain close to a target state 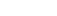 in which both populations function within their dynamic range. The variables 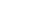 and 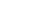 evolve as
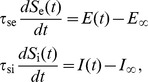
(6)where 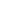 and 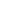 determine the time scales of the adaptation process for the excitatory and the inhibitory population. We can consider equation (5) in steady state at the time scale of adaptation since the adaptive process described by equation (6) is much slower than the population dynamics that are governed by equation (5). The steady state solution of (5) is located at the intersection of the nullclines 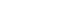 and 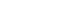. The nullclines are given by
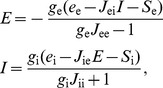
(7)where the activities 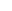 and 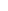 are strictly positive. The explicit steady state solution reads
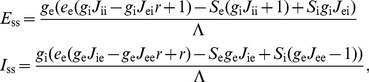
(8)where 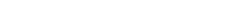 and 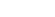. Stability of this solution requires that the matrix of coefficients of equation (5) has eigenvalues with a negative real part. This is guaranteed when
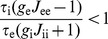
(9)

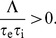
(10)


We now assume that conditions (9) and (10) are satisfied and thus that equation (5) has a stable solution. We can then use the steady state values of equation (8) and substitute these in equation (6) to formulate the evolution of the adaptation variables 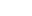 and 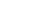 as
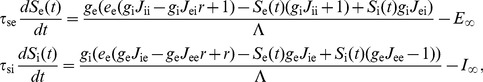
(11)We now want to determine the stability conditions for the solution of equation (11). Stability of the solution, where both 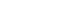 and 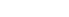, requires that perturbations from the solution decay to zero. Using the same approach as above, we can derive the stability conditions:
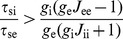
(12)

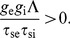
(13)Since we assume that condition (10) holds and we only allow positive gains, condition (13) is always fulfilled. However, condition (12) imposes extra restrictions on the stability of a recurrent network with HSE. This condition will determine the stability of the adaptation dynamics and, thus, whether the time-averaged activity state of the network will approach the target state 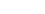. Since we assumed that condition (9) holds, we know that
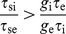
(14)is a sufficient condition to fulfill inequality (12) and to guarantee stability of the HSE controlled network.
